# Description of four new synallactid species (Holothuroidea, Synallactida, Synallactidae) from the tropical Western Pacific Ocean

**DOI:** 10.3897/zookeys.1231.142729

**Published:** 2025-03-13

**Authors:** Yunlu Xiao, Ning Xiao

**Affiliations:** 1 Department of Marine Organism Taxonomy and Phylogeny, Institute of Oceanology, Chinese Academy of Sciences, Qingdao 266071, China; 2 Institute of Deep-sea Science and Engineering, Chinese Academy of Sciences, Sanya 572000, China; 3 University of Chinese Academy of Sciences, Beijing 100049, China; 4 Laboratory for Marine Biology and Biotechnology, Qingdao Marine Science and Technology Center, Qingdao, China; 5 Shandong Province Key Laboratory of Experimental Marine Biology, Institute of Oceanology, Chinese Academy of Sciences, Qingdao 266071, China

**Keywords:** *Amphigymnas*, *Bathyplotes*, new species, ROV, seamounts, *Synallactes*, taxonomy

## Abstract

Four synallactid specimens were collected during four deep-water benthic fauna surveys (2013–2018) conducted at three seamounts (‘Jiaolong’, ‘Y3’, and ‘M4’) and the Ganquan Plateau in the South China Sea, tropical Western Pacific Ocean, at depths ranging from 344 to 3610 m. Morphological examination of these specimens revealed four new species belonging to three genera, which are described as *Synallactestenuibrachius***sp. nov.**, *Bathyplotesliaoi***sp. nov.**, *Bathyplotesvaricolumna***sp. nov.**, and *Amphigymnasganquani***sp. nov.** Notably, *Amphigymnasganquani***sp. nov.** represents the first report of an *Amphigymnas* species in the South China Sea. Detailed descriptions are provided for the morphological features, including the type locality, and geographic and bathymetric distributions of these species. These findings contribute to the understanding of seamount biodiversity and provide valuable insights for advancing research on seamount ecology, management, and conservation.

## ﻿Introduction

Holothuroids, commonly known as sea cucumbers, are echinoderms from the class Holothuroidea. They play a critical role in shaping benthic community structure and biogeochemical processes through feeding, fecal production, and locomotory activities (Roberts et al. 2001; Witbaard et al. 2001). Currently, more than 1800 accepted holothuroid species are recognized (WoRMS 2024a), distributed across various marine environments from shallow water to hadal zones (Iken et al. 2001; Jamieson et al. 2011; Lee et al. 2017). The majority of these species are found in the tropical Indo-West Pacific region (Conand 1990). The current classification system for the class Holothuroidea was established by Miller et al. (2017), who conducted extensive molecular phylogenetic analyses of representatives of most families and species-level taxa. Their findings supported the existence of seven orders: Apodida, Dendrochirotida, Elasipodida, Holothuriida, Molpadida, Persiculida, and (Synallactida. Within the order (Synallactida, three families are recognized: the predominantly shallow-water Stichopodidae Haeckel, 1896, and the deep-water Synallactidae Ludwig, 1894, and Deimatidae Théel, 1882. The family Synallactidae is one of the least studied large taxa of deep-sea holothuroids (Solís-Marín 2005; Gebruk et al. 2012; Solís-Marín et al. 2024). It is almost exclusively found in deep-sea habitats (Solís-Marín 2003) and comprises more than 80 accepted species across ten genera (*Allopatides* Koehler & Vaney, 1905; *Amphigymnas* Walsh, 1891; *Bathyplotes* Östergren, 1896; *Capheira* Ludwig, 1893; *Dendrothuria* Koehler & Vaney, 1905; *Galatheathuria* Hansen & Madsen, 1956; *Paelopatides* Théel, 1886; *Pseudothuria* Koehler & Vaney, 1905; *Scotothuria* Hansen, 1978; *Synallactes* Ludwig, 1894). This variable family is characterized by the absence of tentacle ampullae, a paired gonad, and ossicles composed basically of tables and rods (Solís-Marín 2003, 2005; O’Loughlin and Ahearn 2005). Although new species have been frequently described in recent years, the family Synallactidae remains poorly understood (Thandar 2008; Massin and Hendrickx 2010; O’Loughlin et al. 2013; Samyn and Vandenspiegel 2016; Solís-Marín et al. 2024). This persistent deficiency in taxonomic research presents a significant obstacle to advancing our understanding of the diversity and ecological roles within this family.

Seamounts are unique deep-sea landforms that play an important role in shaping marine biodiversity patterns and processes (Boehlert and Genin 1987; Hannington et al. 1995). They are “diversity hotspots”, often hosting higher species richness than other coastal and oceanic areas (Worm et al. 2003; Samadi et al. 2006; Morato et al. 2008, 2010) and serving as important habitats for benthic invertebrates (Pitcher and Bulman 2007). The tropical Indo-West Pacific region, as the world marine biodiversity center, is one of the priority regions for future research on seamount ecosystems and biodiversity (Xu 2021). This region contains numerous seamounts such as the Yap (Y3), Mariana (M2), and Caroline (M4) seamounts. The South China Sea (**SCS**), a semi-enclosed marginal sea in the tropical Western Pacific Ocean, includes many seamounts scattered across its abyssal plain (Huang et al. 2023). Although seamounts are acknowledged as highly productive biodiversity hotspots (Clark et al. 2012; Wagner et al. 2020; Yesson et al. 2021), research on these ecosystems remains limited, and reports of Synallactidae from seamounts are particularly rare. To bridge the knowledge gap, the Institute of Oceanology, Chinese Academy of Sciences (**IOCAS**) conducted several scientific expeditions between 2013 and 2018. During these surveys, four synallactid specimens were collected from three seamounts (named ‘Jiaolong’, ‘Y3’, and ‘M4’) and the Ganquan Plateau in the SCS, at depths ranging from 344 to 3610 m, using the Remotely Operated Vehicle submersible (**ROV**) ‘Faxian’ (discovery in Chinese) and the manned submersible ‘Jiaolong’ and ‘Shenhaiyongshi’. Detailed examination of ossicles and external morphological features revealed that the four specimens belonged to three genera and represent four new species, which are described and illustrated in this work.

## Materials and methods

**Figure 1.**
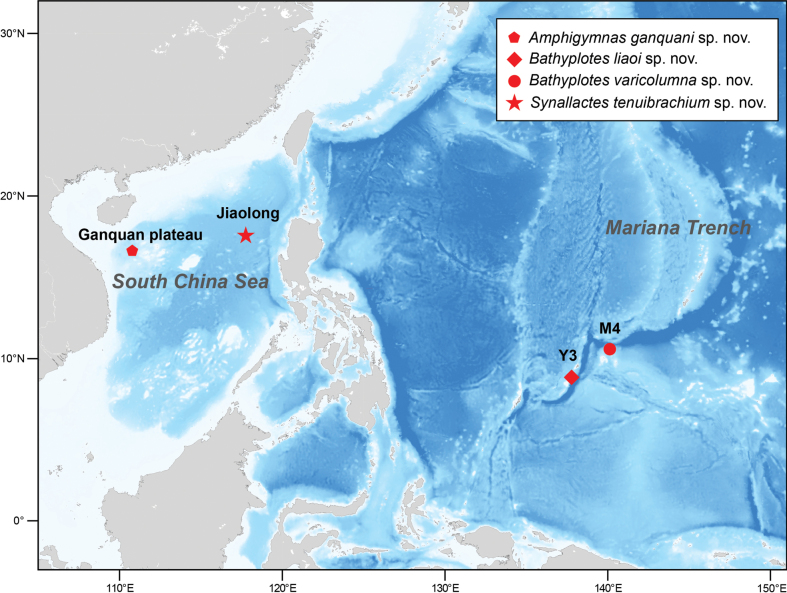
Sampling sites of the studied holothuroid species from tropical Western Pacific, showing the location of three seamounts.

The Remotely Operated Vehicle submersible (**ROV**) ‘Faxian’, deployed from the research vessel R/V ‘Kexue’ (science in Chinese), along with the Human Occupied Vehicles (**HOVs**) ‘Jiaolong’ and ‘Shenhaiyongshi’, were used to collect four synallactid specimens during four surveys conducted in the tropical Western Pacific (2013–2018) (Fig. 1). These specimens were photographed immediately on board prior to preservation. They were then fixed in 95% ethanol for morphological examination and subsequently stored in the Marine Biological Museum of Chinese Academy of Sciences (**MBMCAS**) at Qingdao, China. Due to the limited availability of molecular data for Synallactidae, identification of the four specimens relied solely on morphological characteristics.

Morphological observations were performed using a stereomicroscope (ZEISS Stemi 2000-C, Wetzlar, Germany). To prepare ossicles, soft tissues from the body wall, tentacles, papillae, and tube feet were digested in a solution of 15% sodium hypochlorite for 4–5 h. The digested samples were then rinsed with distilled water, air-dried, mounted on aluminum stubs, and coated with gold. The ossicle features of the four specimens were examined and photographed under a Nikon Eclipse Ni-U microscope (Tokyo, Japan) and a Hitachi S-3400N (Tokyo, Japan) scanning electron microscope (**SEM**).

Abbreviations used in the text: **ROV**, Remotely Operated Vehicle submersible; **HOV**, Human Occupied Vehicles; **IOCAS**, the Institute of Oceanology, Chinese Academy of Sciences; **MBM**, Marine Biological Museum of Chinese Academy of Sciences, Qingdao, China; **RN**, registration number; **CN**, collection number; **SCS**, South China Sea; **SEM**, scanning electron microscope.

## Systematics

**Order Synallactida Miller, Kerr, Paulay, Reich, Wilson, Carvajal & Rouse, 2017**

**Family Synallactidae Ludwig, 1894**

### Genus Synallactes

Taxon classificationAnimaliaSynallactidaSynallactidae

Ludwig, 1894

DF86076A-8E6F-5CE6-9DA0-C4D84069FA00

#### Diagnosis.

Body cylindrical or subcylindrical. Tentacles 18–20. Ventral surface flattened, without any marginal border. Ventral tube feet and dorsal papillae in longitudinal series, confined to ambulacra. Three zones of tube feet on ventral surface. Body wall with tri- or quadri-radiate tables, the distal ends of the arms with a larger or smaller number of perforations, often lateral processes forming a complex lattice-like network with similar processes of other arms. Spire consisting of a single pillar, terminally divided or perforated, or both [adapted from Solís-Marín 2005: 570–571].

#### Type species.

*Synallactesalexandri* Ludwig, 1894.

#### Type locality.

North Pacific Ocean, Azuero Peninsula, Gulf of Panama, depth 588 m (Solís-Marín 2003).

### Synallactes tenuibrachius sp. nov.

Taxon classificationAnimaliaSynallactidaSynallactidae

4AD8E6FC-48BE-5724-A7A2-EC01B548452C

https://zoobank.org/B4D25768-197C-41A8-8363-7D3EB2AB17FF

Figs 2 , 3

#### Material examined.

***Holotype*** • West Pacific, the Jiaolong Seamount in the SCS, depth 3610 m, 5 Jul. 2013, preserved in 95% ethanol, CN: III, RN: MBM286921.

#### Diagnosis.

Body subcylindrical, convex dorsally, flattened ventrally. Mouth and anus terminal. Dorsal papillae conical, scattered on the bivium, with alternating large and small papillae. Ventrolateral papillae placed in a single row on each body side. Midventral radius with one or two small tube feet at the anterior and posterior ends. Ventrolateral tube feet arranged in double alternate rows. Dorsal body wall perforated plates and tables, tables with four or five slender arms. Papillae with tables, spires of tables higher than those of tables in the dorsal body wall. Ventral body wall tables with four or five arms. Tables with 4–6 arms in ventral tube feet, the distal ends of the arms enlarged, branched, rarely connected, and possessing a number of perforations.

#### Description.

**Figure 2.**
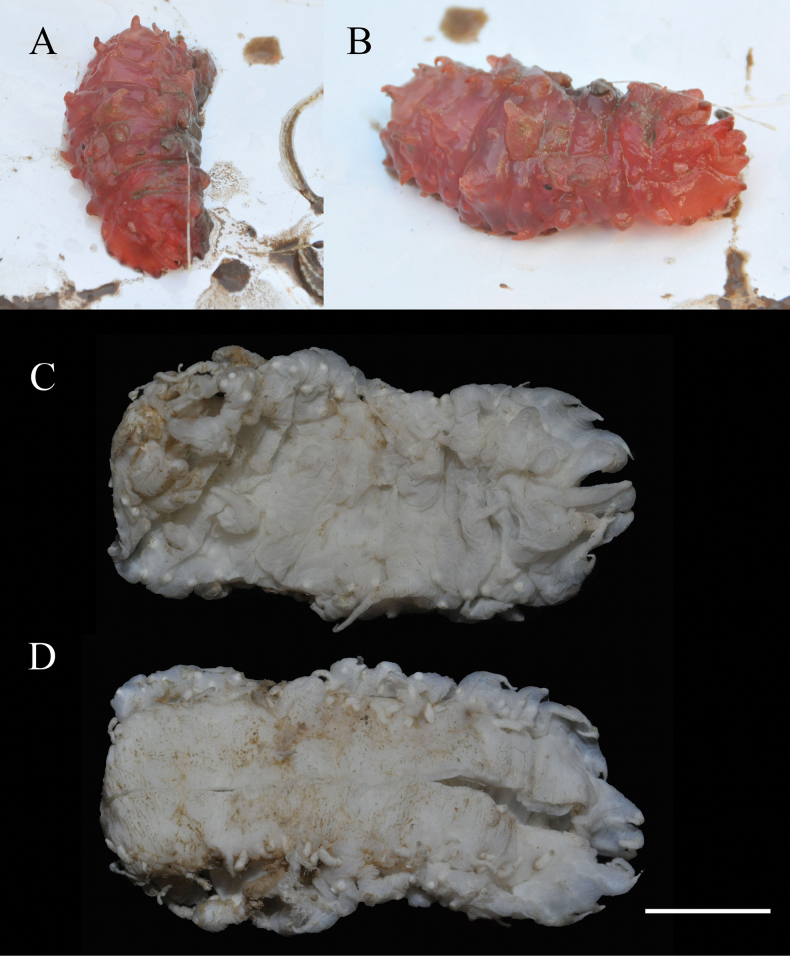
*Synallactestenuibrachius* sp. nov. holotype: MBM286921 **A, B** living specimen **C, D** preserved specimen **C** dorsal view **D** ventral view. Scale bar: 2 cm.

**Figure 3.**
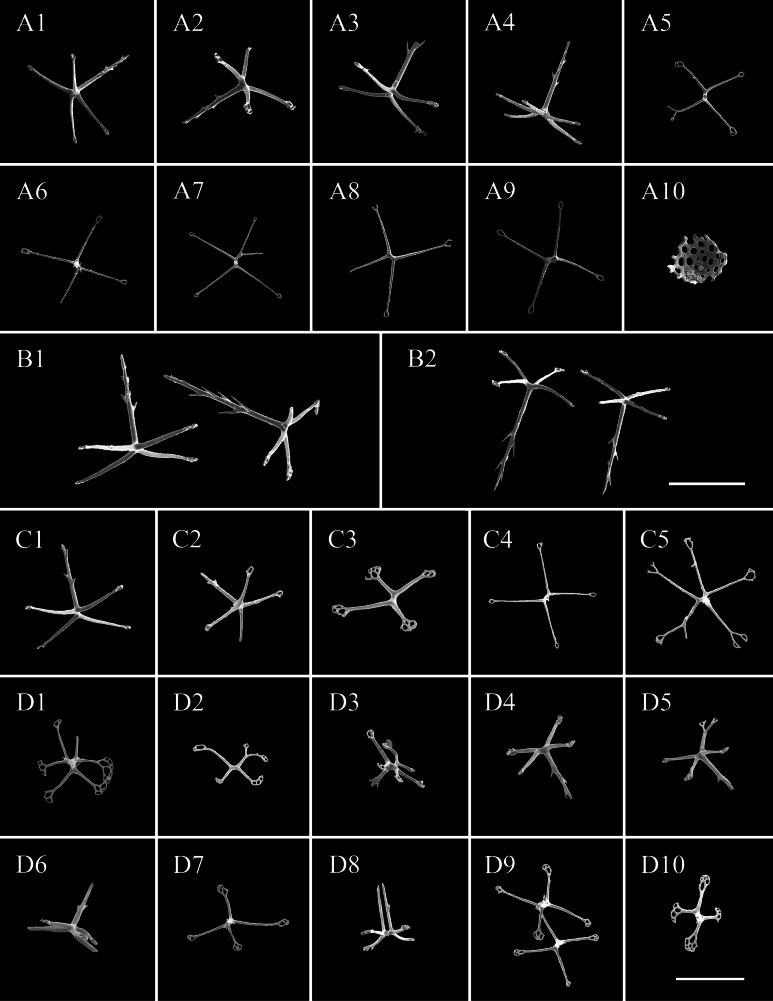
SEM images of *Synallactestenuibrachius* sp. nov. holotype: MBM286921. Ossicles from **A1–A10** dorsal body wall **B1–B2** papillae **C1–C5** ventral body wall **D1–D10** ventral tube feet. Scale bars: 100 μm.

Body subcylindrical, slightly convex dorsally, flattened ventrally. Skin soft and gelatinous. Color red in life (Fig. 2A, B). After 95% ethanol fixation, color white, body length 9 cm and width 4 cm (Fig. 2C, D). Mouth and anus terminal. Tentacles, calcareous ring, and polian vesicles lost. Dorsal surface with numerous large and small papillae, scattered all over the bivium (Fig. 2A–C). Large papillae arranged in longitudinal rows along dorsal radii, ~ 10 in each row; small papillae mainly distributed in both dorsal interradii, ~ 20 on each body side (Fig. 2C). Ventrolateral papillae large, forming a simple row around the margin of the brim (Fig. 2A, B). Ventrolateral tube feet conical, arranged in a double alternate row, ~ 22 on each body side (Fig. 2D). Midventral tube feet radius with one or two small tube feet at the anterior and posterior ends of the body (Fig. 2D). ***Ossicles*.** Dorsal body wall tables with four or five arms (Fig. 3A1–A9), arms slightly broader at the tip with one or two perforations, 50–70 μm in length, spires with simple tips, often bearing several spines on the side, 82–100 μm in height; simple concave perforated plates (Fig. 3A10), 75 μm in diameter. Papillae with four-armed tables (Fig. 3B1, B2), the spires significantly higher than those in the dorsal body wall, 110–155 μm in height. Ventral body wall with tables similar to those in the dorsal body wall (Fig. 3C1–C5), except that the end of arms bifurcated or bearing more perforations (up to 5) in some tables. Tables with 4–6 arms in ventral tube feet (Fig. 3D1–D10), irregular in shape, the distal ends of the arms enlarged, branched, and possessing several perforations, sometimes the enlarged ends of the arms connected with one another (Fig. 3D1); rarely a four-armed table with two parallel pillars (Fig. 3D8), ~ 68 μm in height, without transverse beam.

#### Type locality.

The Jiaolong Seamount in the South China Sea, tropical Western Pacific, depth 3610 m.

#### Etymology.

The name of the new species is a combination of the Latin words *tenuis* meaning ‘slender’ and *brachium* meaning ‘arm’. It refers to the very slender arms of tables in the body wall.

#### Distribution.

Only known from its type locality.

#### Remarks.

The new species, *Synallactestenuibrachius* sp. nov., is characterized by the tube feet arranged in a double alternate row along each ventrolateral radius and all tables possessing very slender arms. Among the five species of *Synallactes* with single-pillared tables featuring pointed tops (i.e., *S.aenigma*, *S.horridus*, *S.robertsoni*, *S.profundus*, and *S.laguardai*; Solís-Marín 2003; Solís-Marín 2005), the new species most resembles *S.aenigma* Ludwig, 1894, *S.horridus* Koehler & Vaney, 1905, and *S.profundus* (Koehler & Vaney, 1905) based on the ossicle features of the body wall. Specifically, all have single-pillared tables with pointed tops and four- or five-armed discs, with the distal ends of the arms exhibiting only a few perforations. However, there are some differences between them. Compared with *S.aenigma*: 1) In the new species, the midventral ambulacrum bears only one or two small tube feet at the anterior and posterior ends of the body. In contrast, Ludwig (1894) noted that *S.aenigma* possesses a few tube feet organized into two long rows on the midventral radius. 2) The anus in *S.tenuibrachius* sp. nov. is terminal, whereas in *S.aenigma*, it is dorsal. 3) The spires in *S.tenuibrachius* sp. nov., are generally unbranched at the end, with some arms having enlarged ends that may connect to each other, while in *S.aenigma*, the spires of tables are divided into 2–10 elongated tips at the end, and the arms of tables never seem to be connected. 4) *Synallactestenuibrachius* sp. nov. differs from *S.aenigma* by the types of ossicles in the tube feet. The tube feet of *S.tenuibrachius* sp. nov. have only tables, whereas those of *S.aenigma* have spiny, curved support rods, and well-developed mesh-like discs in addition to tables.

Compared with *S.horridus*: 1) *S.tenuibrachius* sp. nov. exhibits numerous large and small papillae scattered on the dorsal surface and only one or two small tube feet placed on the midventral ambulacrum at the anterior and posterior ends of the body. By contrast, *S.horridus* has large conical papillae on all radii except midventral radius, and some tube feet are confined to the anterior region of midventral radius. 2) The body wall of *S.tenuibrachius* sp. nov. contains tables with cross-shaped discs and small perforated plates, but no rods, while the body wall of *S.horridus* has many cruciform bodies and rods, with perforated plates absent. 3) All the arms and spires of tables in the body wall of *S.tenuibrachius* sp. nov. are more slender and fragile, compared to the solid and high spires of the cruciform bodies in the body wall of *S.horridus*.

Compared with *S.profundus*: The new species differs from *S.profundus* by the arrangement and number of the ventral tube feet. The tube feet in *S.tenuibrachius* sp. nov. are arranged in a double alternate row on each ventrolateral radius, while the midventral radius is completely naked. In *S.profundus*, the tube feet are arranged in a single row on each ventrolateral radius, with the midventral radius hosting a few tube feet near the anterior and posterior ends.

### Genus Bathyplotes

Taxon classificationAnimaliaSynallactidaSynallactidae

Östergren, 1896

89A098C4-0556-514F-972D-0505242DCBB9

#### Diagnosis.

Tentacles 15–20. Mouth ventral, anus dorsal, subdorsal or nearly terminal. Skin rather thick. Body with sole-like ventral side, usually with marginal appendages. Midventral ambulacrum with a few tube feet or naked. Ventrolateral tube feet arranged in a single row or multiple rows. Dorsal papillae more or less distinctly arranged in rows. Tables with cross-shaped disc and a spire formed by 1–5 pillars, usually with several transverse beams (rarely without transverse beams); C- shaped bodies may be present [adapted from Solís-Marín 2003: 124].

#### Type species.

*Bathyplotesnatans* (Sars, 1868) by original designation.

#### Type locality.

Lofoten, northern Norway, depth 366–549 m.

### Bathyplotes varicolumna sp. nov.

Taxon classificationAnimaliaSynallactidaSynallactidae

FAE422C6-6426-522C-B71E-AE9315D6ACD9

https://zoobank.org/7E4D63FA-EB18-4906-96BB-57E8B25315B8

Figs 4 , 5

#### Material examined.

***Holotype*** • West Pacific, the M4 Seamount located in the Caroline Ridge, Dive FX-Dive137 (10°35.04'N, 140°07.27'E), depth 1195 m, 21 Aug. 2017, preserved in 95% ethanol, CN: C144, RN: MBM286925.

#### Diagnosis.

Body elongated, ventrally flattened. Mouth ventral, anus terminal. Peltate tentacles 16. Dorsal surface with scattered large papillae and laterally with a simple row of small papillae. The dorsal side irregularly arranged with several low ‘fungiform’ whitish warts. Ventral surface with scattered minute tube feet. Dorsal body wall lacking ossicles. Papillae cross-shaped tables and rods, tables with 4–7 arms, 3–5 pillars, and without transverse beams. Cross-shaped tables with single pillars in low whitish warts. Cross-shaped tables with single pillars, support rods, and irregular shaped ossicles in the ventral body wall. Cross-like discs of tables, perforated plates, and support rods in ventral tube feet. Rods, smaller tables, and irregular deposits in tentacles.

#### Description.

**Figure 4.**
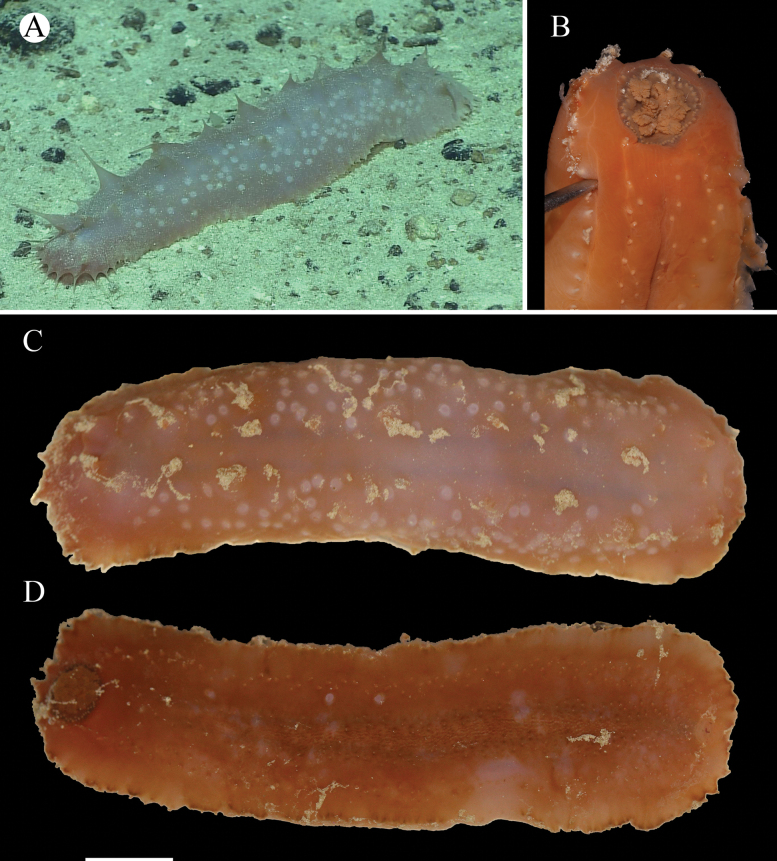
*Bathyplotesvaricolumna* sp. nov. holotype: MBM286925 **A** in situ image **B** tentacles **C** dorsal view **D** ventral view. Scale bar: 3 cm.

**Figure 5.**
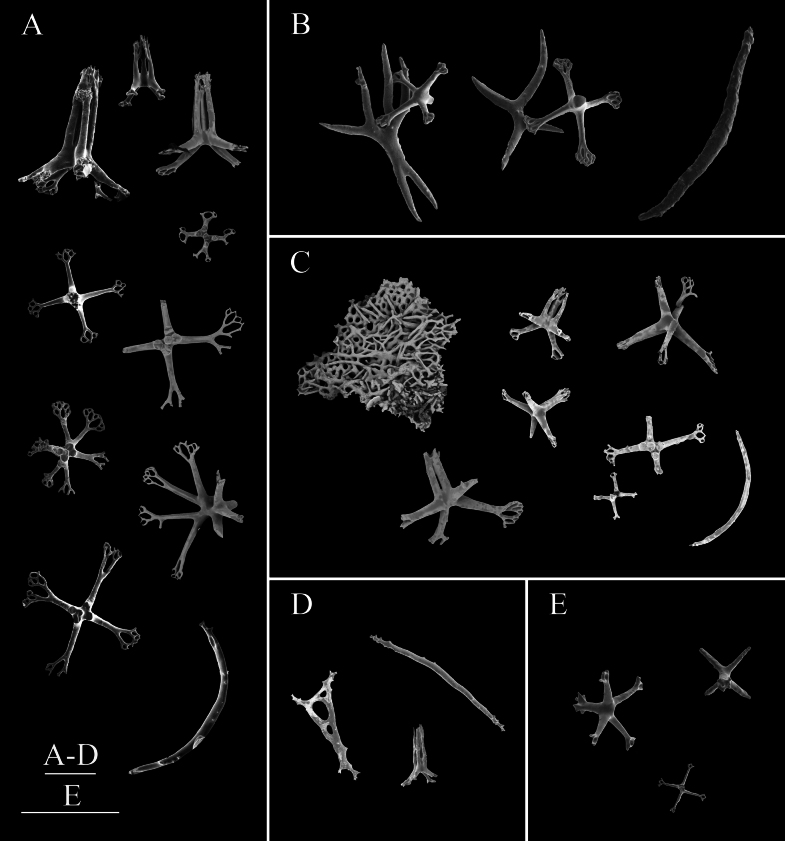
SEM images of *Bathyplotesvaricolumna* sp. nov. holotype: MBM286925. Ossicles from **A** dorsal papillae **B** ventral body wall **C** ventral tube feet **D** tentacles **E** ‘fungiform’ whitish warts. Scale bars: 100 μm.

Body elongated, ventrally flattened, body wall thin and pliable. Color pale pink in situ, and orange in life (Fig. 4A, B). Body 24 cm long and 6.5 cm wide in living specimen (Fig. 4C, D). Mouth ventral, anus terminal. Peltate tentacles 16. Circum-oral papillae present (Fig. 4B). Bivium with some scattered large papillae and laterally with a simple row of small papillae (Fig. 4A, C). A number of low whitish warts arranged along the two dorsal interradii (Fig. 4C). Trivium with some scattered minute tube feet (Fig. 4B, D). Ventral surface with three series (a middle series and one lateral series on each side) of tube feet (Fig. 4D), the middle series forming multiple rows, occupying a third of the ventral surface, other two series forming two or three rows on each side. Brim narrow and retracted, formed by ventrolateral papillae (Fig. 4A, B). ***Ossicles*.** Dorsal body wall lacking ossicles. Dorsal papillae with tables and rods (Fig. 5A), cross-shaped discs of tables 190–260 μm across, with 4–7 arms, each arm ~ 45–155 μm long, the ends of the arms enlarged and pierced with holes, the spires composed of 3–5 pillars, without transverse beams, the top of pillars fused, making the tip of spires spinous; rods curved, 280–315 μm long. Ventral body wall with tables, rods and irregularly shaped ossicles (Fig. 5B), cross-shaped discs of tables with four arms, and only one central pillar (often truncated); rods up to 95 μm. Cross-like discs of tables in ventral tube feet 100–222 μm across, with 1–4 pillars, without transverse beams, the top of pillars fused; terminal plates and rods also in tube feet (Fig. 5C). Rods, smaller tables, and irregular deposits in tentacles (Fig. 5D). Tables with four arms in low whitish warts similar to those in the ventral body wall, and possessing only one central pillar, the top of pillars often irregularly branched or truncated (Fig. 5E).

#### Type locality.

The M4 Seamount located in the Caroline Ridge of the tropical Western Pacific, depth 1195 m.

#### Etymology.

The Latin word *varicolumna* means ‘various pillars’ and is used to describe tables characterized by different types of pillars.

#### Distribution.

Only known in its type locality.

#### Remarks.

The body of the new species, with a sole-like ventral side, and its tables featuring cross-shaped discs with typically three or four pillars, confirm its placement within the genus *Bathyplotes*. In most *Bathyplotes* species, tables generally possess four pillars, rarely three or five. However, in the new species, tables exhibit a broader variation, with 1–5 pillars, and 4–7 arms, an uncommon variation within this genus. Among the known species of *Bathyplotes*, the morphology of *Bathyplotesvaricolumna* sp. nov. most closely resembles that of *B.moseleyi*. Both species share tables with numerous arms and dorsal interradii bearing numerous low whitish warts.

Nonetheless, the new species can be distinguished from *B.moseleyi* by the following differences: 1) Both *B.moseleyi* and the new species possess three series of tube feet on the ventral surface: a middle series and one lateral series on each side. However, the middle tube feet in *B.moseleyi* are arranged in a thin double row, whereas the middle tube feet in *B.varicolumna* sp. nov. are scattered irregularly and broadly along the midventral radius. 2) In *B.moseleyi*, tables have discs with 4–8 arms, and their spires are formed by four pillars and one, rarely two transverse beams, or are often entirely devoid of transverse beams. In contrast, the tables in *B.varicolumna* sp. nov. have discs with 4–7 arms, and their spires consist of 1–5 pillars without transverse beams. 3) The tube feet of *B.moseleyi* contain support rods, whereas those of *B.varicolumna* sp. nov. possess tables and terminal plates in addition to rods. 4) In *B.moseleyi*, whitish warts contain only a few rods at their ends. In *B.varicolumna* sp. nov., however, the predominant ossicles are four-armed tables with cross-shaped discs, supported by a single central pillar that is often irregularly branched or truncated at the top.

### Bathyplotes liaoi sp. nov.

Taxon classificationAnimaliaSynallactidaSynallactidae

7C8F86E9-4A92-51B3-8050-F3740D59E862

https://zoobank.org/F8A68557-64F1-41B9-B1C4-8F536D013EDD

Figs 6 , 7 , 8

#### Material examined.

***Holotype*** • West Pacific, the Y3 Seamount near the Yap Trench, Dive FX-Dive 21 (8°51'N, 137°47'E), depth 344 m, 24 Dec. 2014, preserved in 95% ethanol, CN: Y30165, RN: MBM286918.

#### Diagnosis.

Body elongated, dorsally convex, ventrally flattened. Mouth ventral, anus dorsal. Tentacles 18. Dorsal papillae arranged in six longitudinal rows. Brim formed by small ventrolateral papillae. Tube feet irregularly scattered on the ventral surface. Dorsal body wall containing tables with cross-shaped discs, four (rarely five) pillars of tables bearing numerous large spines, and four to six transverse beams; small tables with annular discs; perforated plates. Dorsal papillae with perforated plates, tables with cross-shaped discs, and support rods. Ventral body wall ossicles similar to those in the dorsal body wall, except for some tables with much higher spires. Tube feet with perforated plates, irregularly shaped ossicles, and small tables with annular discs. Tentacles with spiny rods.

#### Description.

**Figure 6.**
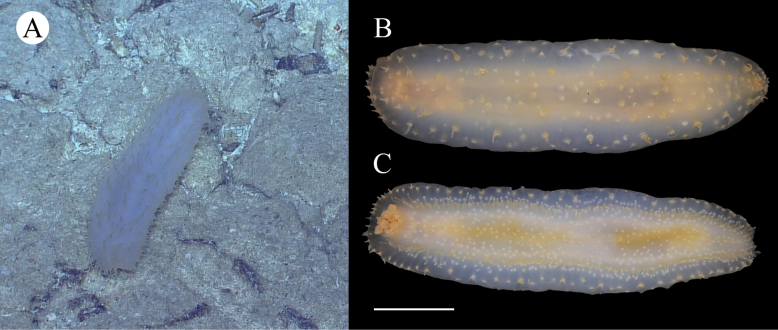
*Bathyplotesliaoi* sp. nov. holotype: MBM286918 **A** in situ image **B** dorsal view **C** ventral view. Scale bar: 3 cm.

**Figure 7.**
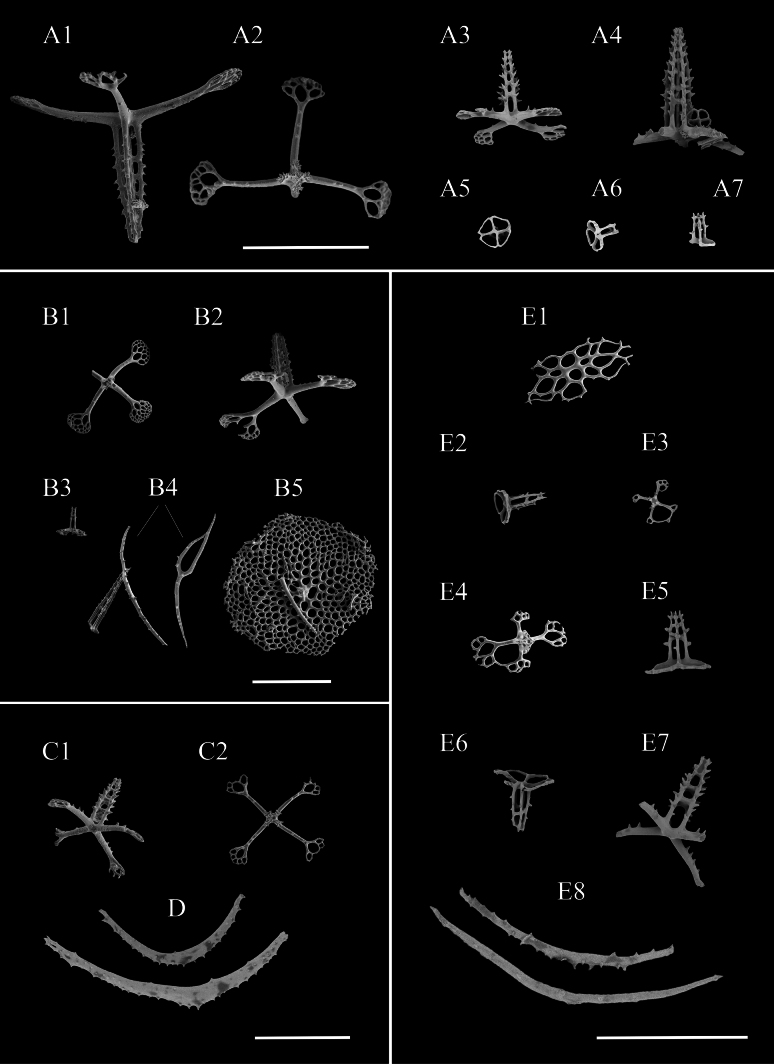
SEM images of *Bathyplotesliaoi* sp. nov. holotype: MBM286918. Ossicles from **A1–A7** dorsal body wall **B1–B5** dorsal papillae **C1, C2** ventral body wall **D** tentacles **E1–E8** tube feet. Scale bars: 200 μm.

**Figure 8.**
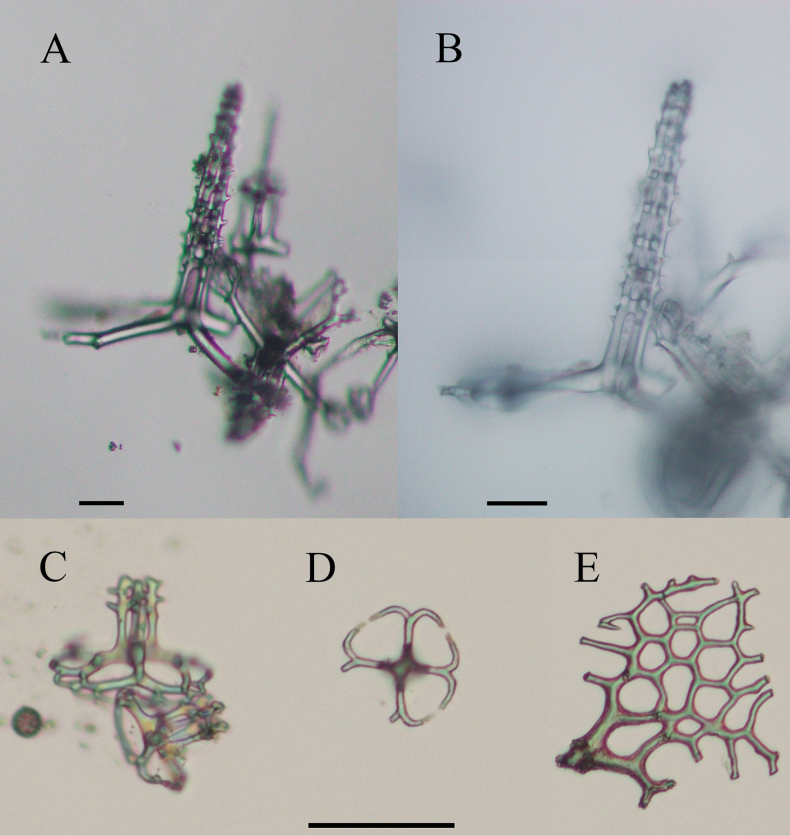
Optical microscope images of *Bathyplotesliaoi* sp. nov. holotype: MBM286918. Ossicles from ventral body wall **A, B** tables with very high spires and four-armed discs **C** small tables with annular discs **D** annular discs **E** perforated plates. Scale bars: 50 μm.

Body elongated, slightly pointed at each extremity, dorsal slightly arched, ventral flattened (Fig. 6A–C). Color in life faint yellow, sub-transparent, dorsal papillae, tube feet, and tentacles darker (Fig. 6B, C). Body 15 cm long and 5 cm wide before fixation (Fig. 6B, C). Mouth ventral, anus dorsal and surrounded by numerous small papillae (Fig. 6B, C). Peltate tentacles 18. Dorsal surface densely covered with approximately equal conical papillae, arranged in six longitudinal rows, with ~ 14–18 papillae in each row (Fig. 6A, B). The jagged edges on both sides of the body formed by a single row of numerous smaller papillae. Tube feet irregularly arranged longitudinally throughout the ventral sole, ~ 86 in each row. ***Ossicles*.** Tables of two types in the dorsal body wall (Fig. 7A1–A7): 1) Cross-shaped discs (Fig. 7A1–A4) with four arms, each arm bearing small spines, 100–200 μm in length, with a number of perforations at the ends, four (rarely five) pillars bearing numerous large spines, four to six transverse beams, the spires measuring 108–200 μm in height, tips formed by fused pillars. 2) Annular discs (Fig. 7A5–A7) with four large holes formed by simple diagonal bars, discs ~ 58 μm in diameter, the spires 30–42 μm high, with one or two transverse beams. Dorsal papillae with most tables (Fig. 7B1–B4) similar to those of the dorsal body wall (first type), rarely smaller tables with four transverse beams (Fig. 7B4); perforated plates, measuring 400 μm in diameter (Fig. 7B5); spinous rods up to 366 μm in length (Fig. 7B4). Ventral body wall tables with four-armed discs, small tables with annular discs, and perforated plates (Fig. 7C1, C2 and Fig. 8), most ossicles of similar features to those in the dorsal body wall, except that a few tables with really high spire, measuring 245 μm in height (Fig. 8A, B). In tentacles (Fig. 7D) rods 70–500 μm long. Tube feet with four types of ossicles: 1) perforated plates (Fig. 7E1); 2) smaller tables with annular discs (Fig. 7E2–E7), ~ 88 μm in diameter, the spires of tables higher than those in the dorsal body wall (second type), measuring 48–64 μm high, four spiny pillars with three transverse beams; 3) rods up to 404 μm in length (Fig. 7E8).

#### Type locality.

The Y3 Seamount near the Yap Trench, tropical Western Pacific, 344 m depth.

#### Etymology.

The species is named after one of the pioneers in the study of holothuroid fauna in China, Professor Yulin Liao.

#### Distribution.

Only known from its type locality.

#### Remarks.

The predominance of four-pillared tables undoubtedly indicates that this species belongs to the *Bathyplotes*. *Bathyplotesliaoi* sp. nov. is mostly similar, in terms of ossicle types, to *Bathyplotespatagiatus* Fisher, 1907, which has been regarded as a synonym of *Bathyplotesnatans*. Both species share the presence of ossicles that include four-armed crosses, small tables with annular discs, and spinous rods. However, the new species can be distinguished from *B.patagiatus* by the presence of tube feet on the midventral radius and differences in the shape of the spires of tables. In *Bathyplotesliaoi* sp. nov., the tables typically have four, rarely five pillars, with their dilated ends of spires usually formed by these fused pillars, rather than expanding into a crown. In contrast, the tables in *B.patagiatus* are robust, with each table possessing four pillars and a broad thorny crown formed by the highly dilated spire ends, resembling a quadrilateral tower. Moreover, the pillar ends in *B.patagiatus* are often unfused. The new species also differs from *B.patagiatus* by the presence of tables with annular discs and five-pillared crosses in the dorsal body wall, as well as perforated plates in the ventral body wall and papillae. In *B.liaoi* sp. nov., the arms of the crosses bear small spines, whereas those in *B.patagiatus* are smooth. The spines on the spires of tables in *B.liaoi* sp. nov. are distributed along the entire length of the pillars, while in *B.patagiatus*, the spines are confined to the upper half, even in tables with exceptionally long spires. In addition, *B.liaoi* sp. nov. lacks C-shaped ossicles present in *B.patagiatus*.

Both *Bathyplotesliaoi* sp. nov. and *B.phlegmaticus* have two types of tables in the body wall: 1) large tables of characteristic form, with cross-like discs and 2) smaller tables with annular discs. The arms and pillars of both types of tables possess spines. However, the new species exhibits several key differences from *B.phlegmaticus*: 1) The number and arrangement of papillae are different. In *B.liaoi* sp. nov.: the dorsal surface was densely covered with conical papillae of approximately equal size, arranged in six longitudinal rows, with ~ 14–18 papillae each row. The jagged edges along both sides of the body were formed by a single row of numerous smaller papillae. In *B.phlegmaticus*, a delicate rim was formed by free papillae, with ~ 35 at the anterior part of the body and ~ 12 along the anterior and posterior lateral edges. Papillae on the ventral side are few, irregularly distributed, and not confined to the radii. 2) Tube feet in *B.phlegmaticus* have rods and a well-developed endplate. However, tube feet in *B.liaoi* sp. nov. include tables with annular discs, irregular large ossicles, rods, and a plate. 3) Tables have different numbers of transverse beams. In *B.phlegmaticus*, the spires of large tables possess 6–8 transverse beams, while smaller tables have 1–3 transverse beams. In *B.liaoi* sp. nov., the large tables have fewer transverse beams (4–6), and the smaller tables have only one or two transverse beams.

### Genus Amphigymnas

Taxon classificationAnimaliaSynallactidaSynallactidae

Walsh, 1891

8A278E91-808F-5D6A-A614-94861A9BA6E2

#### Diagnosis.

Body wall calcareous, brittle, similar to that of the elasipodid family Deimatidae. Mouth ventral, peltate tentacles 20. Dorsal and lateral body with long conical calcareous papillae, including a ventrolateral series. Body flat ventrally, tube feet in ambulacral series or scattered. Anus subdorsal posterior. Ossicles in body wall large table discs with many perforations, discs variably with or lacking spires often comprising three or four pillars, sometimes with more fused pillars, without distal spines or teeth, spires sometimes reduced to short unconnected pillars [adapted from O’Loughlin et al. 2013: 38].

#### Type species.

*Amphigymnasmultipes* Walsh, 1891 accepted as *Amphigymnaswoodmasoni* (Walsh, 1891).

#### Type locality.

Indian Ocean, Andaman Sea, off Dyer Point and North Cinque Island, depth 343–402 m.

### Amphigymnas ganquani sp. nov.

Taxon classificationAnimaliaSynallactidaSynallactidae

63276A58-F401-5F39-988B-8225248F17B5

https://zoobank.org/B0A06953-CB2A-41AD-97D2-295D92330F52

Figs 9 , 10 , 11

#### Material examined.

***Holotype*** • West Pacific, the Ganquan Plateau in the SCS, depth 1350 m, 12 May 2018, preserved in 95% ethanol, CN: GQHT-SY-066-12, RN: MBM286926.

#### Diagnosis.

Body elongated, cylindrical, gradually tapering at both ends. Color yellowish white. Skin fragile, glass-like. Mouth ventral, oral disc with front-suspended dorsal papillae, anus terminal. Peltate tentacles 20. Dorsal papillae arranged in two rows along each dorsal ambulacrum. Ventrolateral papillae arranged in a single row. Ventrolateral tube feet arranged in a single row on each side. Midventral ambulacrum placed in alternate two rows. Dorsal body wall tables with short or high 4-pillared spires and 0–2 transverse beams. Papillae tables and widened midrods, tables with higher spires, four or more usually fused pillars, and three or four transverse beams. Ventral body wall with smaller tables, spires truncate, without transverse beams. Tube feet tables with truncate and high pillars. Tentacles support rods.

#### Description.

**Figure 9.**
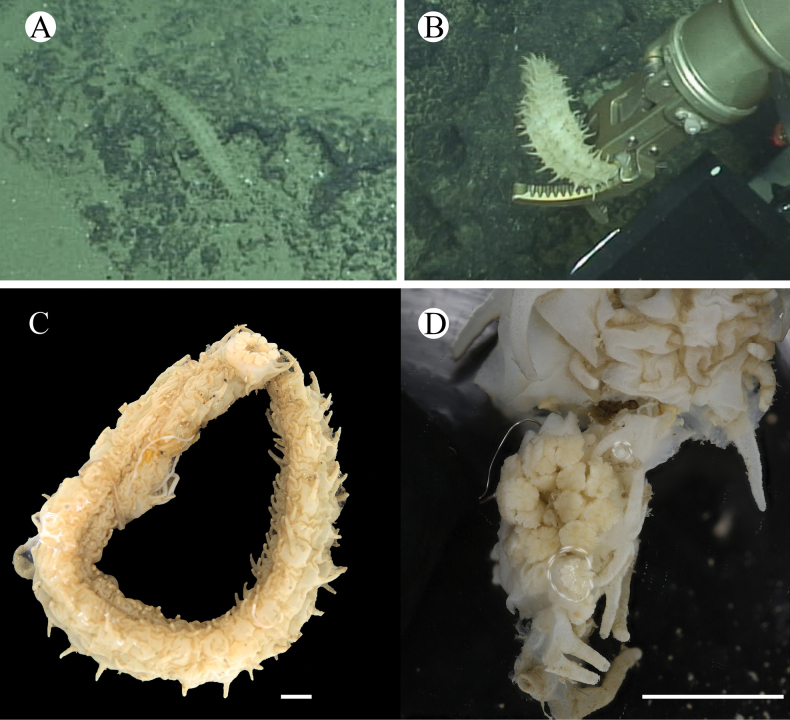
*Amphigymnasganquani* sp. nov. holotype: MBM286926 **A, B** in situ images **C** holotype after fixation **D** tentacles. Scale bars: 1 cm.

**Figure 10.**
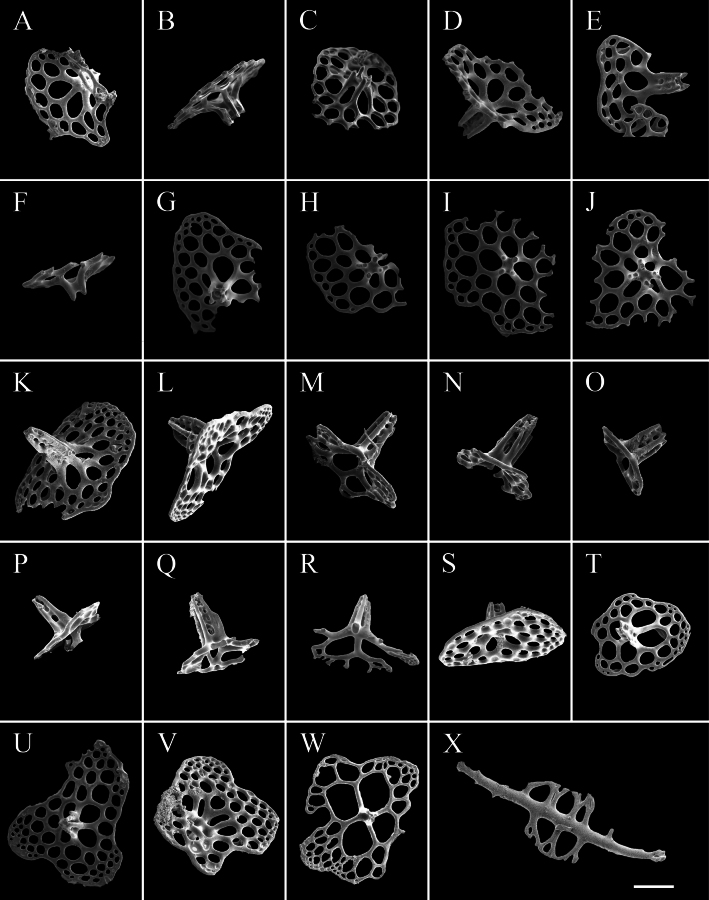
SEM images of *Amphigymnasganquani* sp. nov. holotype: MBM286926. Ossicles from **A–J** dorsal body wall **K–X** dorsal and lateral papillae. Scale bar: 100 μm.

**Figure 11.**
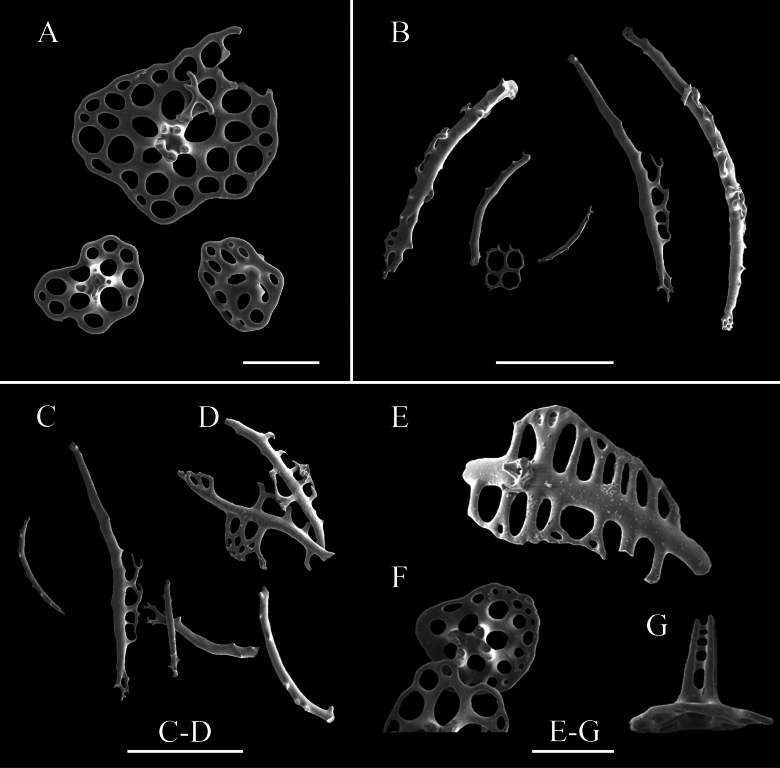
SEM images of *Amphigymnasganquani* sp. nov. holotype: MBM286926. Ossicles from **A** ventral body wall **B** tentacles **C–G** tube feet. Scale bars: 100 μm (**A**, **E–G**); 300 μm (**B–D**).

Body long and cylindrical, slightly slender at both ends (Fig. 9A, B), ventral flattened. Body 28 cm long, 2.5 cm wide mid-body before fixation (Fig. 9C). Color yellowish white, body wall thin, calcareous, and fragile. Mouth ventral, anteriorly overhung by a few dorsal papillae (Fig. 9C, D), anus terminal. Peltate tentacles 20, measuring 0.25–0.3 cm in diameter after fixation (Fig. 9D). Two paired conical papillae placed in a single series on each dorsal radius (four rows across dorsally), each up to 1 cm long after fixation. Ventrolateral papillae arranged in a single row, ~ 22 on each body side, up to 1.5 cm long after fixation. Irregular tube feet relatively large and placed in single rows on ventrolateral radii, ~ 60 in each row. Midventral tube feet small, placed in alternate two rows, ~ 80 in each row. Longitudinal muscles undivided, tentacle ampullae absent, and gonads arranged in clusters. ***Ossicles*.** Dorsal body wall tables (Fig. 10A–J), discs perforated plates, edges usually incomplete, 220–370 μm across, with numerous perforations of similar size, spires with four pillars (mainly) or more pillars (Fig. 10G, H, J), short or relatively high, lacking or possessing one (rarely) or two transverse beams. Short pillars lacking or with one beam, ~ 50–80 μm in length (Fig. 10A–C, F–J), relatively high pillars with two beams, 100–120 μm in length (Fig. 10D, E). Spires of tables in dorsal and lateral papillae possessing much higher and firmer spires (Fig. 10K–W) than those in dorsal body wall, discs up to 400 μm across, with four large central perforations and many small outer perforations, four or more pillars, up to 150 μm in length, usually fused, making the number of transverse beams uncertain, some spires of tables possibly possessing 3–4 transverse beams; widened midrods in papillae up to 600 μm long, with marginal projections (Fig. 10X). Ventral body wall with smaller tables (Fig. 11A), discs 144–267 μm across, edges more complete and smooth, spires truncate, without transverse beams. Tentacles predominantly rods, curved, bearing irregular marginal projections, up to 803 μm long (Fig. 11B). Tube feet support rods straight to curved, widened midrods bearing irregular marginal projections, some placing through the entire length (Fig. 11C–E); tables with truncate pillars (Fig. 11F), without transverse beams; tables with high pillars, possessing four beams (Fig. 11G).

#### Type locality.

The Ganquan Plateau in the South China Sea, tropical Western Pacific, depth 1350 m.

#### Etymology.

The specific name refers to the Ganquan Plateau, where the holotype was sampled.

#### Distribution.

Currently known from the type locality.

#### Remarks.

The new species belongs to *Amphigymnas* Walsh, 1891, characterized by the glass-like body wall formed by numerous large tables, large conical papillae on the dorsal and ventrolateral radii, and a midventral row of small tube feet filled with many support rods.

The new species differs from the *A.bahamensis* Deichmann, 1930 by the arrangement of ventral tube feet and the number of pillars of dorsal tables. Dorsal tables in the new species have at least four pillars, whereas those of *A.bahamensis* have rare 3-pillared truncate spires. Additionally, the ventral tube feet in the new species are arranged along both the ventrolateral and midventral radii, while those in *A.bahamensis* are restricted to the midventral radius. The external morphological differences between the new species and *Amphigymnaswoodmasoni* are slight. However, the new species can be distinguished primarily by ossicle features: Tables in *A.woodmasoni* have four large central perforations surrounded by many smaller peripheral holes, and the spires of tables are usually rather primitive and reduced to four spines (without forming pillars) on the body walls and papillae. In contrast, the spires of tables on body walls and papillae in the new species are relatively high and often exhibit four or more pillars, rather than being reduced to spines. Notably, in the papillae they can reach heights of up to 150 μm. In comparison to *Amphigymnasstaplesi* O’Loughlin in O’Loughlin et al. 2013, the new species exhibits several distinguishing characteristics: The oral disc of *A.staplesi* is surrounded by a continuous series of papillae, whereas the new species has only front-suspended dorsal papillae. The new species is quite different from *A.staplesi* by the ossicle features: 1) In *A.staplesi*, the tables of dorsal body wall have discs with four large central perforations and many smaller outer perforations, and the spires have four pillars and two transverse beams. In the new species, the dorsal tables have discs with perforations of approximately equal diameters, and the spires have four or more pillars and 0–2 transverse beams; 2) The pillars of the new species are relatively higher, sturdier, and often irregularly fused, distinguishing them from those in *A.staplesi*; 3) Additionally, the new species possesses support rods, which are absent in *A.staplesi*; 4) The new species lacks endplates in its ventrolateral tube feet, whereas *A.staplesi* possesses endplates.

The genus *Amphigymnas* was newly recorded in the SCS, and the discovery of the new species expands the geographical distribution of this genus.

## Discussion

Until recently, a total of ten genera and 85 accepted species in the family Synallactidae (WoRMS 2024b). Using distribution data from published literature and findings from this study, we examined the geographical distribution and species diversity of synallactids across the world’s oceans, with a particular focus on the tropical Western Pacific. Table 1 presents a summary of the distribution of synallactid species in the Pacific Ocean, with a focus on the tropical Western Pacific.

**Table 1.** Overview of the distribution of synallactid species in the Pacific Ocean and tropical Western Pacific.

Genus	Number of accepted species	Number of species in the Pacific Ocean	Species in the tropical Western Pacific	
			Number	Species
*Allopatides*	2	1	1	*A.corrugatus*
*Capheira*	2	0	0	–
*Dendrothuria*	2	1	1	*D.megalopharynx*
*Galatheathuria*	1	1	1	*G.aspera*
*Paelopatides*	21	7	3	*P.appendiculata*, *P.illicitus*, and *P.ovalis*
*Pseudothuria*	1	0	0	–
*Scotothuria*	1	0	0	–
*Synallactes*	27	15	9	*S.chuni*, *S.discoidalis*, *S.gilberti*, *S.heteroculus*, *S.monoculus*, *S.multivesiculatus*, *S.nozawai*, *S.sagamiensis*, and *S.triradiate*
*Bathyplotes*	25	13	9	*B.angustus*, *B.cinctus*, *B.crebrapapilla*, *B.dofleinii*, *B.imperfectus*, *B.moseleyi*, *B.natans*, *B.punctatus*, and *B.sulcatus*
*Amphigymnas*	3	1	1	*A.woodmasoni*
Total	85	39	25	–

In summary, more than half of the species within each genus described occur in the Pacific Ocean, with 29 synallactid species (including the four new species described here) identified in the deep water of the tropical Western Pacific. This represents over one-third of all species in the family Synallactidae, highlighting the higher synallactid species richness of this region. However, only three species (i.e., *Synallactestenuibrachius* sp. nov., *Amphigymnasganquani* sp. nov., and *Galatheathuriaaspera*) have been reported in South China Sea. This underscores the need for further deep-sea biodiversity surveys in the SCS to better understand the diversity, distribution patterns, and ecological characteristics of holothuroid fauna in this underexplored region. Such studies will contribute to global marine biodiversity knowledge, support regional conservation strategies, and inform ecosystem-based management approaches for the tropical Western Pacific.

## Supplementary Material

XML Treatment for Synallactes

XML Treatment for Synallactes tenuibrachius

XML Treatment for Bathyplotes

XML Treatment for Bathyplotes varicolumna

XML Treatment for Bathyplotes liaoi

XML Treatment for Amphigymnas

XML Treatment for Amphigymnas ganquani

## Additional information

### Author ORCIDs

Yunlu Xiao https://orcid.org/0009-0005-9787-033X

### Data availability

All of the data that support the findings of this study are available in the main text.
